# Know Your Body Through Intrinsic Goals

**DOI:** 10.3389/fnbot.2018.00030

**Published:** 2018-07-03

**Authors:** Francesco Mannella, Vieri G. Santucci, Eszter Somogyi, Lisa Jacquey, Kevin J. O'Regan, Gianluca Baldassarre

**Affiliations:** ^1^Institute of Cognitive Sciences and Technologies, National Research Council - CNR, Rome, Italy; ^2^Laboratoire Psychologie de la Perception (UMR 8242), Paris Descartes - CPSC, Paris, France

**Keywords:** developmental robotics, developmental psychology, intrinsic motivations, goals, body

## Abstract

The first “object” that newborn children play with is their own body. This activity allows them to autonomously form a sensorimotor map of their own body and a repertoire of actions supporting future cognitive and motor development. Here we propose the theoretical hypothesis, operationalized as a computational model, that this acquisition of body knowledge is not guided by random motor-babbling, but rather by autonomously generated goals formed on the basis of intrinsic motivations. Motor exploration leads the agent to discover and form representations of the possible sensory events it can cause with its own actions. When the agent realizes the possibility of improving the competence to re-activate those representations, it is intrinsically motivated to select and pursue them as goals. The model is based on four components: (1) a self-organizing neural network, modulated by competence-based intrinsic motivations, that acquires abstract representations of experienced sensory (touch) changes; (2) a selector that selects the goal to pursue, and the motor resources to train to pursue it, on the basis of competence improvement; (3) an echo-state neural network that controls and learns, through goal-accomplishment and competence, the agent's motor skills; (4) a predictor of the accomplishment of the selected goals generating the competence-based intrinsic motivation signals. The model is tested as the controller of a simulated simple planar robot composed of a torso and two kinematic 3-DoF 2D arms. The robot explores its body covered by touch sensors by moving its arms. The results, which might be used to guide future empirical experiments, show how the system converges to goals and motor skills allowing it to touch the different parts of own body and how the morphology of the body affects the formed goals. The convergence is strongly dependent on competence-based intrinsic motivations affecting not only skill learning and the selection of formed goals, but also the formation of the goal representations themselves.

## 1. Introduction

The first “object” that newborns start to play with is their own body, in particular by engaging with self-touch activities. Body activity starts in the fetus at 8 weeks of gestation with spontaneous movements called General movements (Piontelli et al., 2014). These movements continue to be part of the motor activity of infants during their first months of life but gradually more controlled movements become dominant (Thelen, 1995). This controlled motor activity (Piontelli et al., 2014) continues for many years after birth (Bremner et al., 2008) and presumably determines the formation of a “body schema” (Rochat and Striano, 2000), a sensorimotor map and a repertoire of actions that constitute the core of future cognitive and motor development.

The importance of self-touch activity for infants is supported by empirical evidence. Infants after birth react differently to external touch events compared to self-touch events: for example, Rochat and Hespos (1997) found that head-turning in response to a tactile stimulation in the mouth area was three times more frequent when the stimulation was externally produced than self-produced, thus showing the unique status of self-touch for infants. Moreover, it seems plausible to consider self-touching as a self-sufficient activity: for instance, we do not need to include vision as part of the sensory input that determines early self-touch events. This is justified first by the very poor use of vision by fetuses in the womb, and second by the fact that infants before 10 months of age seem to not use vision to localize external tactile stimulation on their body (Bremner et al., 2008; Ali et al., 2015).

In this work we propose the theoretical hypothesis, operationalized as a computational model, that early body knowledge in infants is not acquired through random motor-babbling, but guided by self-generated goals, autonomously set on the basis of intrinsic motivations (IMs). The concept of IMs was introduced in animal psychology during the 1950s and then extended in human psychology (Berlyne, 1950, 1960; White, 1959; Deci and Ryan, 1985; Ryan and Deci, 2000) to describe a set of motivations that were incompatible with the Hull's theory of drives (Hull, 1943) where motivations were strictly connected to the satisfaction of primary needs. Different experiments (e.g., Harlow, 1950; Montgomery, 1954; Kish, 1955; Glow and Wtnefield, 1978) showed how exploration, novel or surprising neutral stimuli and even the possibility to affect the environment can modify the behavior of the agents, thereby driving the acquisition of knowledge and skills in the absence of tasks directly required for biological fitness. Further neurophysiology research (e.g., Chiodo et al., 1980; Horvitz, 2000; Redgrave and Gurney, 2006) showed how IMs can be linked to neuromodulator activity, and in particular to dopamine. These results highlighted the role of IMs in enhancing neural plasticity and driving the learning of new skills (Mirolli et al., 2013; Fiore et al., 2014).

Following biological inspiration, IMs have also been introduced in machine learning (e.g., Barto et al., 2004; Schmidhuber, 2010) and developmental robotics (e.g., Oudeyer et al., 2007; Baldassarre and Mirolli, 2013) to foster the autonomous development of artificial agents and the open-ended learning of repertoires of skills. Depending on their functions and mechanisms, different typologies of IMs have been identified (Oudeyer and Kaplan, 2007; Barto et al., 2013; Santucci et al., 2013) and classified broadly into two main groups (Baldassarre et al., 2014): (1) knowledge-based IMs (KB-IMs), divided into (1a) novelty based IMs related to novel non-experienced stimuli, and (1b) prediction-based IMs, related to the violation of the agent's predictions; and (2) competence-based IMs (CB-IMs) related to action, i.e., to the agent's competence to change the world and accomplish self-defined *goals*. While in their first implementations in computational research KB-IMs and CB-IMs were indistinctly used to drive autonomous skill acquisition (e.g., Schmidhuber, 1991; Oudeyer et al., 2007), different authors underlined how the signal generated by CB-IMs has to be preferred when developing agents that has to learn to accomplish new tasks (Oudeyer and Kaplan, 2007; Santucci et al., 2012; Mirolli et al., 2013). In particular, while KB-IM mechanisms generate learning signals based on the acquisition of knowledge, for example based on the improvement of a forward model of the world, CB-IM mechanisms generate learning signals based on the acquisition of competence, for example based on the capacity of achieving a certain desired state (e.g., the capacity of an inverse model or of a state-action controller to achieve a goal state).

Based on these insights, authors started to use CB-IMs for autonomous skill acquisition (Barto et al., 2004; Oudeyer et al., 2007; Schembri et al., 2007a,b; Hart and Grupen, 2011; Santucci et al., 2014b; Kompella et al., 2015). Recent research has started to use CB-IMs for the autonomous generation and/or selection of *goals* which can then drive the acquisition of skills (Merrick, 2012; Baldassarre et al., 2013; Baranes and Oudeyer, 2013; Santucci et al., 2016) and the optimization of learning processes in high-dimensional action spaces with redundant robot controllers (Rolf et al., 2010; Baranes and Oudeyer, 2013; Forestier and Oudeyer, 2016). The present research has been developed within the CB-IM framework, and particular the model presented here uses competence measures to select goals. In line with empirical and computational perspectives (Balleine and Dickinson, 1998; Russell and Norvig, 2003; Thill et al., 2013; Mannella et al., 2016), and also with most works reviewed above, here goals are intended as agent's internal representations of a world/body state or event (or of a set of them) having these properties: (a) the agent can internally activate the representation of the goal even in the absence of the corresponding world state or event; (b) the activated goal representation has the power of focussing the behavior of the agent toward the accomplishment of the goal and to generate a learning signal when the world state matches the goal (“goal-matching”).

Given the connection between CB-IMs and goals, in this paper we present a new hypothesis where these two elements play an important role in the early phases of body knowledge acquisition, i.e., in the first months after the infant's birth. In particular, under our hypothesis the initial infant's exploration determines the formation of proto-representations of sensory events. As soon as the baby discovers the possibility of re-activating those proto-representations a CB-IM signal for obtaining those specific sensory events is generated. This signal improves the information about the current competence (probability of obtaining a sensory event given an action) and the discovered events become intrinsic goals that guide both the learning and the selection of the motor commands to achieve them. Importantly, under the presented hypothesis this “goal-matching” signal also modulates the encoding and consolidation of the outcome representations themselves so that the learning processes defining sensory encoding and motor control are coupled together into an integrated sensorimotor learning system.

Do we really need the notion of *goal* to account for the development of body knowledge? An important alternative hypothesis might rely on the direct use of IMs to drive the acquisition of stimulus-response behavior, in particular on the basis of trial-and-error behaviors (Sutton and Barto, 1998; Mannella and Baldassarre, 2007; Caligiore et al., 2014; Williams and Corbetta, 2016), which are model-free reinforcement learning strategies, strengthened by intrinsic rewards. We shall not here be evaluating such possible alternatives, since the primary purpose of this paper is to fully articulate and operationalize the goal-directed hypothesis, which is a model-based reinforcement learning framework, for future simulation studies and empirical tests. Nevertheless it should be said that the goal-directed hypothesis challenges other hypotheses based on stimulus-response/trial-and-error learning processes for at least two reasons. First, the *learning* of multiple actions (e.g., to touch different body parts) relying on a stimulus-response mechanism seems to require *different stimuli* able to trigger those different actions. In this respect, actions that allow an infant to touch different parts of own body would *start from the same sensory state* (touch, sight, proprioception, etc.). Their acquisition thus seems to require some internally generated patterns/stimuli to which to link them: we hypothesize these patterns/stimuli are represented by different goals (*goal-based learning*, Baldassarre et al., 2013). The use of model-free strategies alone cannot guide behavior in conditions in which the environment does not give enough information to make a choice while model-based solutions allow decision making through the use of the information stored within an internal model. Second, once the infant has acquired those different actions she should be able to *recall* a specific one at will, independently of the current sensory and body state: again model-free reinforcement learning would seem to not allow this whereas internally activated goals could allow it (*goal-based action recall*). In the discussion (section 4) we will consider the differences between our model and other goal-directed approaches.

In this paper we present our hypothesis, implementing a computational model that allows us to investigate the details of the proposed theory and provide quantitative measures that could be useful for future experimental validation. The model is used as a controller (sections 2.2 and 4) for a simulated planar robot composed of a torso and two kinematic 3DoF arms exploring its own body in a 2D environment (section 2.1). Sensory information from self-touch activity is used by the system to form goals and drive skill learning. Results of the tests of the model are presented (section 3) together with their possible implications for ongoing empirical experiments with human infants (section 3.3). Section 4 presents a detailed description of the model equations. The final section of the paper (section 5) discusses relevant related literature and possible future development of the presented model.

## 2. The model

This section describes the functioning and learning mechanisms pivoting on goals that allow the model to autonomously acquire knowledge on own body.

### 2.1. Agent's body

**Figure 1:**
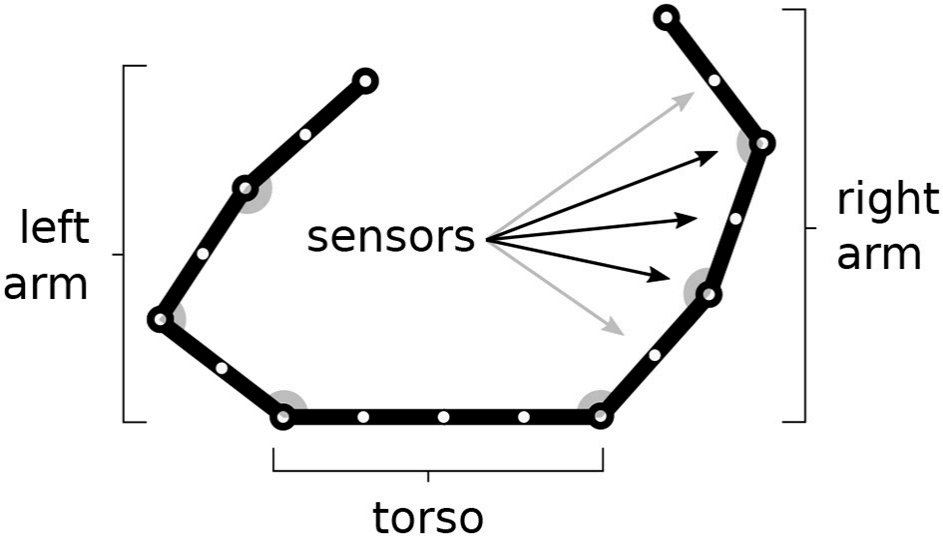
The simulated agent's body with two 3-DoF kinematic arms and a torso. 30 touch sensors are equally distributed over the whole body (here fewer sensors have been represented).

The model is tested within a simulated body living in a two-dimensional space. The body is formed by two arms each formed by 3 links attached to a “torso” (Figure 1). The resulting 6 degrees of freedom (DoF) of the body receive motor commands from the model and as a consequence perform movements. The movements are simulated by only considering kinematics (changes of the joint angles) and no dynamics (the body does not have an inertia).

The body is covered by 30 touch sensors that can activate when touched by a “hand.” Note how the sensors, that are uniformly distributed over the body, belong to a one-dimensional space. The activation of sensors is caused by the two “hands” (arm end-points), in particular the sensor activation is computed on the basis of the sum of two Gaussian functions each getting as input the distance of the sensor from respectively the two hands. Sensors that are nearby the extremity of one “hand,” including the one on the end-point itself, are only sensitive to the other hand to avoid their permanent activation.

The simulation is divided into trials. Each trial ends after a fixed time interval has elapsed or when the agent reaches the selected goal (“goal-matching” event, see section 2.2).

### 2.2. Overview and core aspects of the model functioning and learning

Section 4 illustrates the functioning and learning of the model in mathematical detail. Instead, this section overviews such aspects at a level of detail sufficient to understand the results presented below.

**Figure 2:**
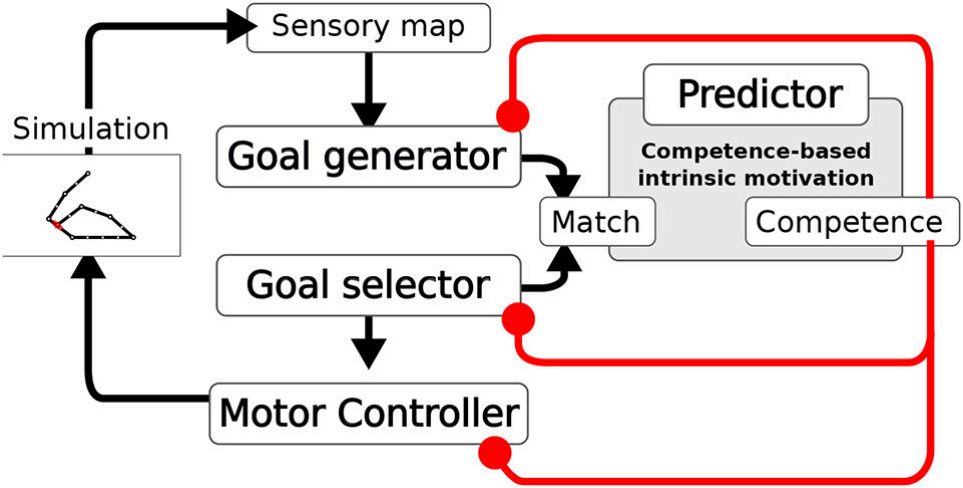
Architecture of the model. A Sensory map encodes the touch sensations. The Goal Generator learns to abstract the sensory information (action outcomes). The Goal Selector selects the goals to pursue. The Motor Controller generates the movements. Based on the information on whether the activities of the two layers match, the measures of competence and competence improvement are computed. These measures modulate the learning of the Goal Generator, the Goal Selector selection, and the learning of the Motor Controller.

The system is composed of four main components (Figure 2): the Goal Generator, the Goal Selector, the Motor Controller and the Predictor. The Goal Generator is responsible for the autonomous generation of the mapping from the domain of sensory input patterns to the domain of internally encoded representations. These representations encode possible states of the world, in particular outcomes of actions, that can be later internally activated as goals. In particular, the Goal Generator receives as input the positive *change* of the activation of the touch sensors distributed over the body. This change is encoded into two-dimensional patterns where the first dimension represents the spatial location of sensors on body, and the second represents the sensor activation amplitude (see section 4 for details). The Goal Generator then performs an unsupervised clustering of the perceived changes by using a self-organizing neural map (SOM). Each output unit of the SOM learns to respond to sensory input patterns that best fit with the prototype stored in the afferent weights of that unit. The output layer of the SOM tends to preserve in its topology the similarity present in the sensory input space: units that are closer to each other in the SOM output layer acquire prototypes that correspond to patterns that are closer to each other in the sensory input space. The unsupervised learning process driving the online clustering in the Goal Generator, which takes place at each time step of the simulation, is modulated by a measure of the current competence based on the prediction for the occurrence of an *action-outcome contingency*. This contingency is detected internally as a match between the sensory encoded representations (the outputs of the SOM) triggered at any time step of the trial, and the goal representation, internally activated from the begin of each trial. The competence measure is computed by the Predictor and is further described later in this section. The higher the competence prediction related to a given goal, the lower is the learning rate of the update of the related outcome prototype. Moreover, the higher the average competence prediction of all stored goals, the lower is the learning rate of all prototypes. This two-fold modulation tends to freeze an outcome prototype when the related goal is accomplished with more reliability and when the system becomes able to accomplish all discovered goals.

The Goal Selector is formed by a vector of units, corresponding one-to-one to the SOM output layer units, that localistically encode goals. At the beginning of each trial, the component selects the goal to be pursued by means of a softmax function. The resulting output is a one-hot vector with the winning unit switched on. The input to the softmax function used to decide the winning unit is based on the difference (error) between the competence prediction for each goal, given by the Predictor, and the actual goal-outcome match. In particular, a decaying average of such error is used. Thus, also the goal selection is modulated by the current agent's competence.

The Motor Controller is composed of three components: a dynamic-reservoir recurrent neural network (Jaeger, 2001; Jaeger et al., 2007; Mannella and Baldassarre, 2015), a random trajectory generator and an associative memory. The dynamic-reservoir is a recurrent network whose dynamics is regulated by the goal received as input from the Goal Selector. The random trajectory generator outputs a trajectory at each trial based on a sinusoidal oscillator with a randomly-chosen setting of its parameters. Both the read-out units of the recurrent network and the output of the random trajectory oscillator contribute to control the two arms, with the competence for the currently chosen goal defining their relative importance weight. The Motor Controller is trained by means of a novel model-based reinforcement learnig algorithm exploiting the goal-based reward. The algorithm relies on two processes: (1) The associative memory stores and updates the end-point posture for each goal based on the occurrence of goal-outcome contingencies; (2) The end-point postures stored in the associative memory are then used as models to train the readout of the recurrent network. In particular, the current chosen goal recalls the end-point posture to which it is related in the associative memory, and the readout units of the recurrent network are trained to acquire an attractor dynamics corresponding to that end-point posture. The learning in the associative memory is also guided by the competence for the currently chosen goal. When the competence for a goal is low the learning rate for the update of the relative end-point posture is high, while the more the competence for that goal gets higher the more the learning rate for the update of the relative end-point posture gets lower. Overall, when competence is low the random generation of motor trajectories prevails. Meanwhile, goal-outcome contingency events lead the learning of the end-point postures. As competence gets higher the learning processes are slowed down and the exploitation of the so far learned readout of the recurrent network prevails in defining the motor trajectories. When the agent eventually achieves the maximum competence for a goal the related motor skill is frozen.

As we have seen, all the learning processes within the system depend on the detection of goal-outcome matches corresponding to external action-outcome contingencies. This detection is based on the fact that the units of the generated goal and those of the selected goal have a one-to-one correspondence. In particular, when a couple of corresponding outcome unit and selected-goal unit co-activate a *goal-outcome matching* signal is delivered to the whole system. The CB-IMs that guide the exploration and the learning of the system are determined by the activity of the Predictor. This component is a linear neural network that gets as input the activation pattern of the Goal Selector encoding the selected goal, and is trained with a supervised learning algorithm to predict the matching signal for that goal (0 in the case of failure and 1 in case of success). The output of the Predictor represents an esteem of the probability of accomplishing the selected goal. This esteem is used to compute two measures of competence. The first measure indicates the system competence for the selected goal, and corresponds to the actual output of the Predictor. The second measure indicates the rate of accomplishment of the selected goal per trial and is given by a decaying average of the error of the predictor trying to predict the goal-outcome matching. These two CB-IM measures modulate the system learning processes.

Summarizing, there are three interacting optimization processes involving three different functions of the system: (1) encoding of action outcomes; (2) motor control learning; (3) competence prediction learning. The optimization of the outcome encoding (1) and the optimization of the motor control (2) are guided by the competence of the agent, which is itself acquired through the optimization of the agent's goal-outcome matching prediction (3).

## 3. Results

This section presents some simulation tests directed to show that the model manages to explore the one-dimensional space of the agent's body and autonomously build knowledge on it. In particular, the autonomous learning processes involving the acquisition of goals and of the motor capabilities to accomplish them under the guidance of contingency detection and competence-based intrinsic motivations converge to a steady equilibrium, thus consolidating the agent's bodily knowledge that allows it to reach at will all different parts of the body with one of the two hands.

The tests involve simulations where the grid of goals (both in the Goal Generator and the Goal Selector layer) is formed by 5 × 5 units (25 possible goals) and the agent's body is uniformly covered by 30 touch sensors. We now illustrate the results of the tests in detail.

### 3.1. Coverage of the body space by the acquired knowledge

We performed 20 different simulations lasting 8,000 trials each. At the beginning of each trial the units in the Goal Selector layer were recruited as representations of a different desired goal, while the units in the Goal Generator layer were triggered by the online encoding of the sensory inputs. The Goal Generator/Goal Selector matching pairs became related to touch events centered in different points in the body space during learning.

We now focus on the analysis of the data referring to one simulation representative of the average performance of the system.

**Figure 3:**
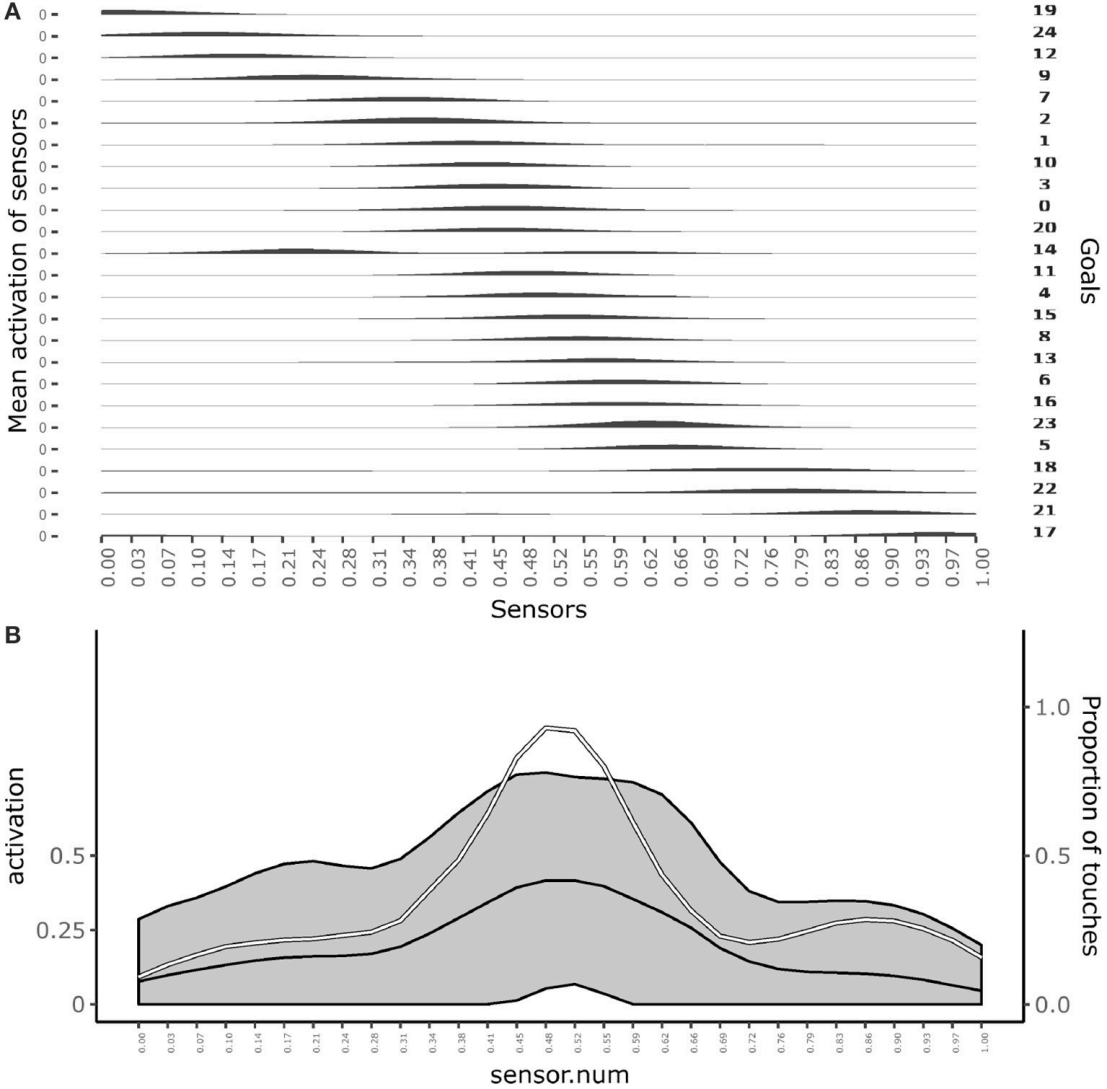
Distribution of touch events. **(A)** At each row of the plot an histogram shows the mean activation of each sensor over the trials where a given goal is chosen. **(B)** Mean activation and standard deviation of each sensor over all the trials after convergence of learning (black curves and gray area). the white curve indicates the touch frequency with random movements. Data collected over 1,000 trials after the learning process stabilized to maximum competence for all goals.

Figure 3A shows which sensors are activated when the different goals are pursued. The figure shows how after learning different goals produce touch events that cover the whole body space. This shows that the goals that the agent forms only partially overlap and also succeed to cover the whole body space.

Figure 3B shows the average activation of sensors over all the learning trials. The figure shows that different parts of the body are touched with different frequencies. In particular, the “chest” area is touched very frequently whereas areas around sensors at distance 0.28 and 0.72 from the left hand (where 1 is the length of the whole body) are touched less frequently. This different frequencies are due to the topology of the agent's body. Random exploration favors the touch of the chest, exposed to reaching of both arms, while disfavors the touch of the “shoulders,” “hidden” in the angle formed by the chest and one arm, and with a medium frequency the rest of the arms, fully exposed to the reaching of the controlateral hand. The touch events activating sensors on the “hands” always involve both of them and so they tend to have peak frequencies.

**Figure 4:**
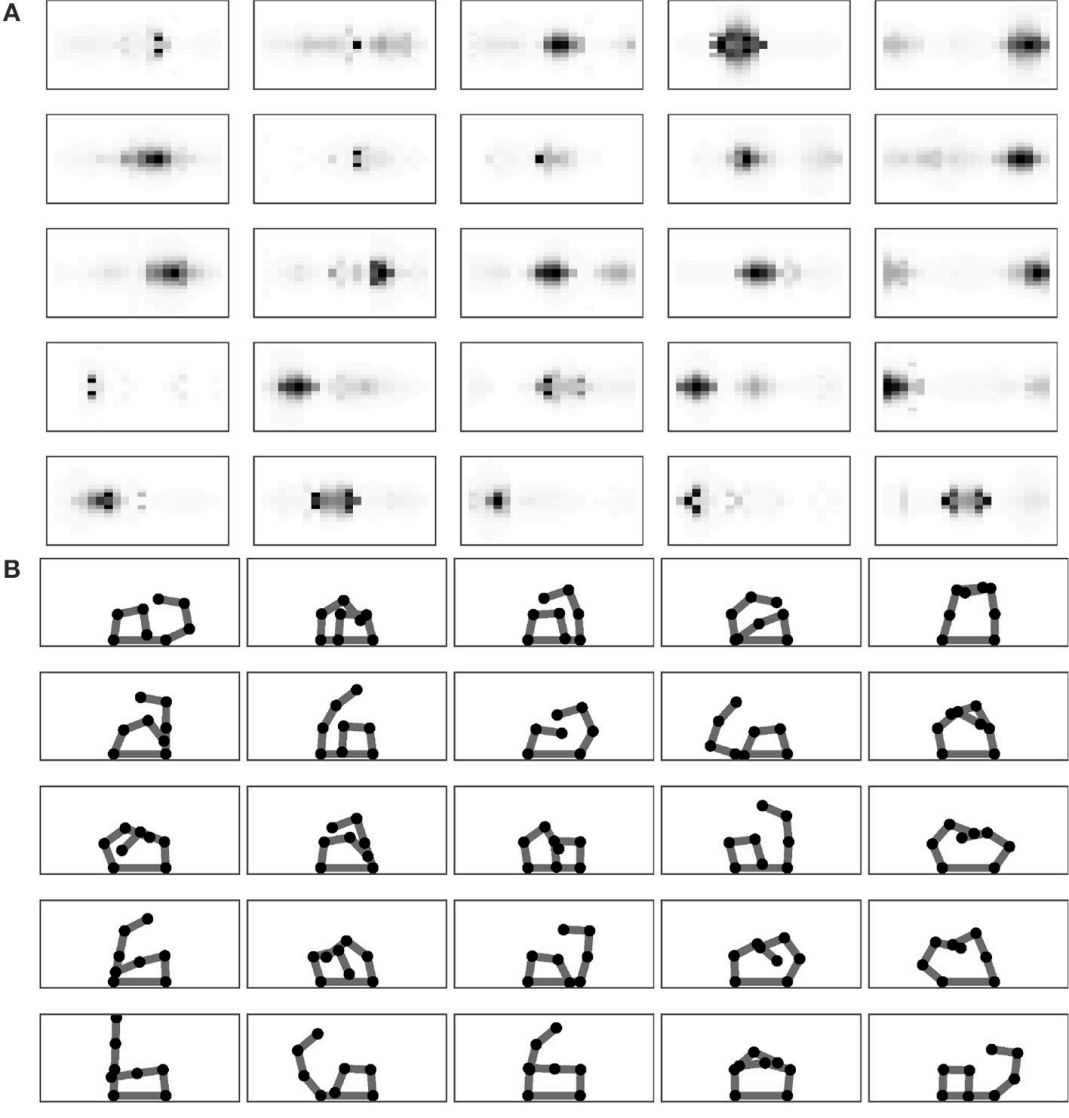
Performance after learning. **(A)** The receptive fields of each goal. **(B)** Target postures related to goals.

Figure 4A analyses the SOM receptive fields related to the different outcomes encoded by the Goal Generator after the completion of the learning process. Recall that each 20 × 20 field represents the activity of a map where the horizontal axis refers to the different sensors located on the one-dimensional body space and the vertical axis refers to the intensity of their activation. Figure 4B shows the posture of the two arms learnt to reproduce the touch event related to each goal. The figures show a tendency of the grid of goals to represent multiple aspects of the touch events. In particular, going from the bottom-left to the top-right of the grid receptive fields tends to represent touch events involving the chest and one arm and then both arms. Instead, the bottom-right dimension of the grid tends to represent touches involving more the left or the right part of the body.

### 3.2. Stability of the acquired knowledge

The system reaches a steady equilibrium guided by the increase of competence for the different goals. **Figure 6** shows how the mean of the competence for each goal, self-estimated by the agent as the prediction to activate the target sensors corresponding to the selected goal (see section 4.1.1.4.), grows until the agent reaches the maximum competence for each goal. At that point, all learning processes regarding body knowledge halt.

The raster plot at the top of the **Figure 6** plots illustrates the positions in time of the matching events (corresponding to the touch of the sensors related to the selected goal). Each row of the raster plot refers to one of the 25 goals. At the beginning of the learning process the system selects different goals and focuses on them until it has properly learnt how to achieve them (see the bottom-left plot of **Figure 6**).

**Figure 5:**
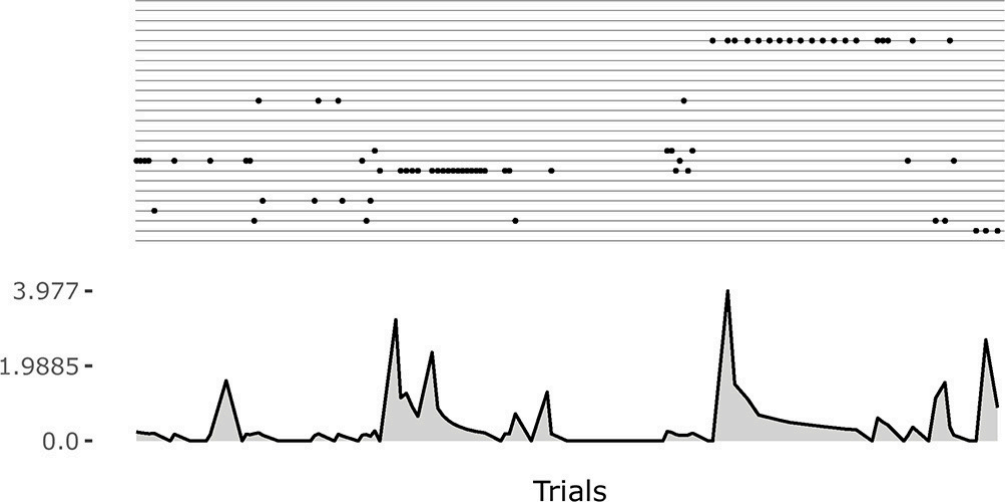
An example of the initial focussing on goals that are just been experienced. On the top a raster plot with each row indicating the match events for a goal. On the bottom a plot indicating the current changes in the weights of the motor controller (Euclidean distance from weights at the previous time step). The initial match event produces a great change in the weights. The following ones refine the motor skill, and the corresponding outcome sensory abstraction, until the competence for the goal is completely acquired.

Note how the system tends to focus on single goals with some persistence after they are discovered (see also Figure 5). This is due to the fact that the competence signal used to select goals changes slowly. This feature turns out to be important for the convergence of the system. Indeed, if one uses a non-smoothed version of the signal (by setting τ_ξ_ = 1 in Equation 20) then the focussing disappears and the system fails to converge. A possible interpretation of this results might be as follows: the focus on a specific goal leads the system to acquire a high competence for that goal; the high competence for the goal stabilizes both the goal representation and the related motor skill; the acquired goals/skills furnish an enough stable “structure” that the system can leverage to build the other goals and skills.

**Figure 6:**
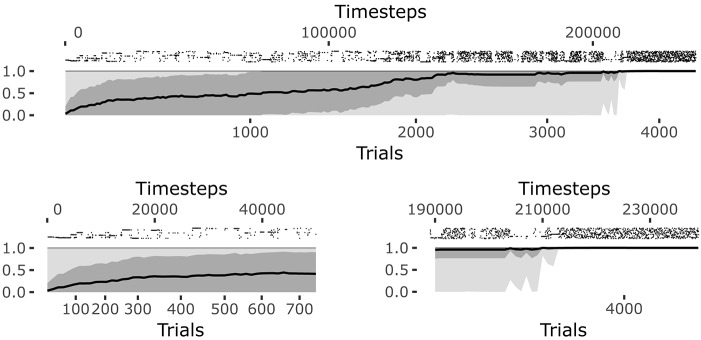
History of goal predictions, indicating the probability of success when pursuing a given goal, during the learning process. Top plot: the black line indicates the average prediction over all the 25 goals, the dark gray shadow indicates the standard deviation, and the light gray shadow indicates the worst and best skill; the raster plot in the upper part shows the matching events for each goal, where different rows correspond to different goals. Bottom plots: zoomed visualization of the initial phase and convergence phase of the learning process, respectively.

When the learning process converges, the system continues to test each goal and the prediction is maintained at its maximum (see the bottom-right plot of Figure 6). At this point all goals start to be equally and randomly selected as they are no more interesting for the system but this, in its current state, must still engage in some activity (see the bottom-left plot of Figure 6). This means the intrinsic motivation and contingency detection capabilities would be ready for the exploration of other sources of knowledge if they were available to the agent.

**Figure 7:**
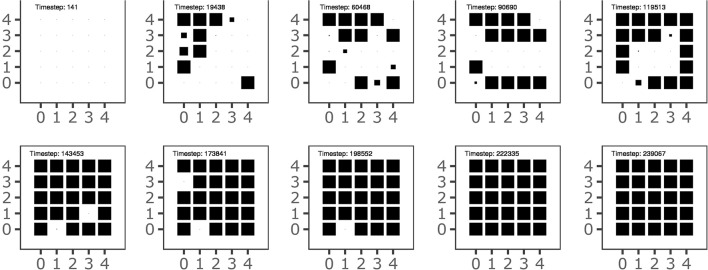
The history of goal formation for the different goals located on the 2-D output map of the goal generator. The area of black squares within each sub-plot is proportional to the system's self-estimated probability of the goal success.

Figure 7 shows the history of the goal formation during the learning process. The figure shows that at different stages of development some goals have been formed but then they are temporary “deleted” and then replaced in the following stages. This indicates that the system searchers an overall goal configuration and motor skill repertoire that settles only when the acquired knowledge covers the whole body space, as shown in Figure 3. The graph also shows that the goals tend to form starting from the outer ring of the map units and then to involve inner units of the map. This might reflect the formation of broad goal categories (and related motor skills) followed by more refined categories. Further investigations are needed to confirm this interpretation.

### 3.3. Features of the behavior of the model

Some peculiar features of the model behavior emerge during its development. These could be possibly compared with the behaviors of children in future empirical experiments.

#### 3.3.1. The time to accomplish the goals diminishes as learning progresses

**Figure 8:**
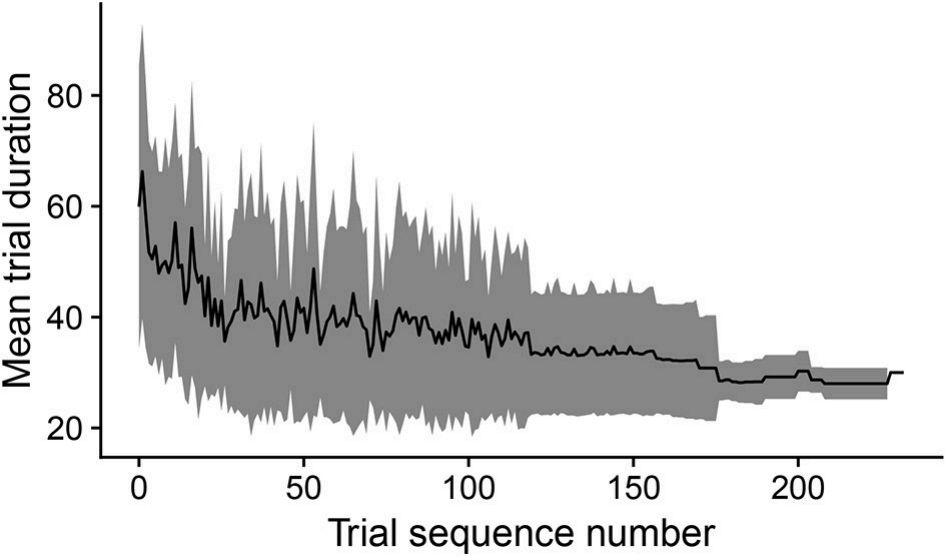
The black line shows the duration of trials, averaged over goals, vs. the cumulated number of successful trials (“matching” events) for each goal. The gray area indicates the standard deviation.

**Figure 9:**
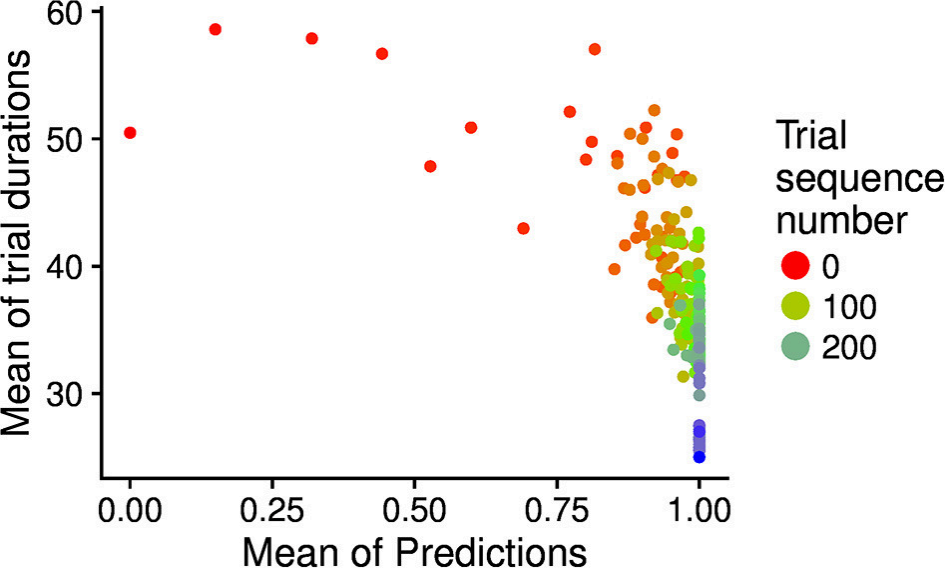
Relation between the “age” of goals (matching event number related to the goal; indicated by the different colors), the mean trial duration (y-axis), and the goal matching prediction (indicating the competence for the goal; x-axis). The graph has been built as follows: for each goal age (also shown in the x-axis of Figure 8), the average trial duration and average prediction over goals were computed and plotted in the graph as one dot.

During development, the time taken by the movements to cause the desired touch sensation set by the different goals progressively decreases. In this respect, Figure 8 shows how the mean trial duration to accomplish the goals actually decreases with the “age” of goals. This reflects the fact that the motor accuracy of the system improves with time dedicated to learn the motor skill of each goal. Figure 9 confirms this interpretation. The figure shows the relation between the time needed to accomplish a goal and the competence of that goal (systems goal-matching prediction probability). The figure shows how lower trial durations are positioned at the bottom-right part of the plot where the value of competence for the goals is very high. The color of the dots, related to the “age” of goals, also indicates that performance time and competence improve with the amount of learning dedicated to each goal. In this respect, note how the competence tends to reach values close to 100% after about 100 successes (matching events). The motor skill, however, continues to increase as shown by the lower trial duration after 200 trials. It might be surprising that motor ability for a goal continues to improve even when the competence-based intrinsic motivation signal becomes low. This is due to the fact that while this signal continues to exert its effect on the selection of the goals, when a goal is selected the related motor skill continues to be trained as much as possible, as it should, by the mechanism driving the echo-state network to produce the goal-related desired posture.

#### 3.3.2. Easy postures are acquired before hard ones

**Figure 10:**
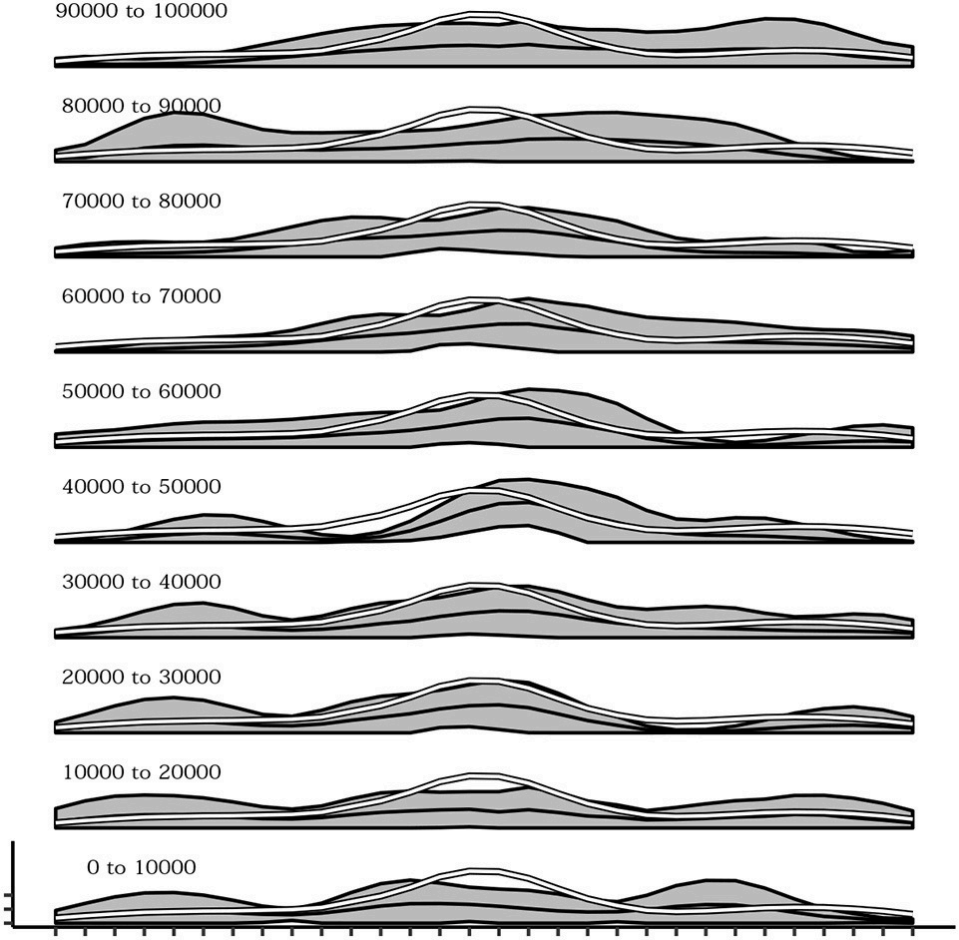
Mean activations of each sensors over several intervals (10,000 timestep) from the beginning (bottom) up to 100,000 timesteps. Sensors belonging to the more easily reachable areas of the agent's body (white line) are the reached more frequently in the initial part of the simulation while later all other areas are reached as well.

During the development of the sensorimotor behavior of the agent there is also a change in which postures are explored. Indeed postures that are easier to be reached due to the physical constraints of the actuators are discovered since the first trials of the simulations while postures that are more difficult to achieve are acquired later on. Figure 10 shows this phenomenon. from bottom to top several plots are presented indicating the mean activation of the touch sensors during different 10,000-timestep-long time intervals. It is evident how during the initial intervals the curve of sensor's activations follows the white line, representing the mean of sensor's activations recorded in a simulation where the agent's motor behavior is kept strictly random. This is an indication that at the beginning of the experiment, postures that are common during random behavior (and thus can be considered less difficult to reach) are more likely to be chosen than others. Instead, going in the top part of the plot series the curve of sensor's activation depart from the white line confirming that the agent is more likely to be focused on postures that are more rare during random behavior (and thus can be considered more difficult to reach).

#### 3.3.3. Areas with more density of sensors are explored before other ones

**Figure 11:**
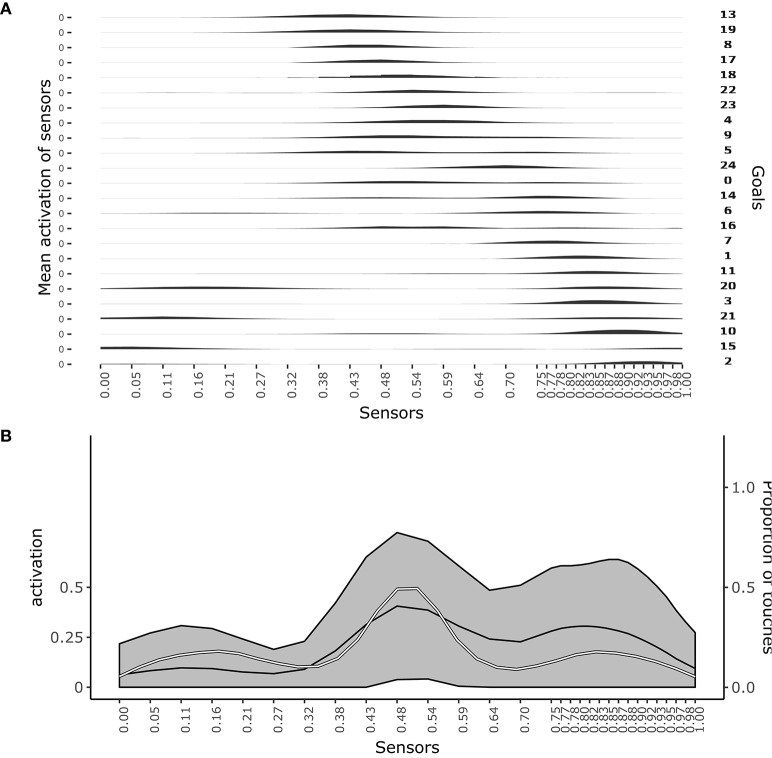
Distribution of touch events in a simulations where the right part of the body has an higher density of sensors. **(A)** At each row of the plot an histogram shows the mean activation of each sensor over the trials where a given goal is chosen. **(B)** Mean activation and standard deviation of each sensor over all the trials after convergence of learning (black curves and gray area). The white curve indicates the touch frequency with random movements. Data collected over 1,000 trials after the learning process stabilized to maximum competence for all goals.

**Figure 12:**
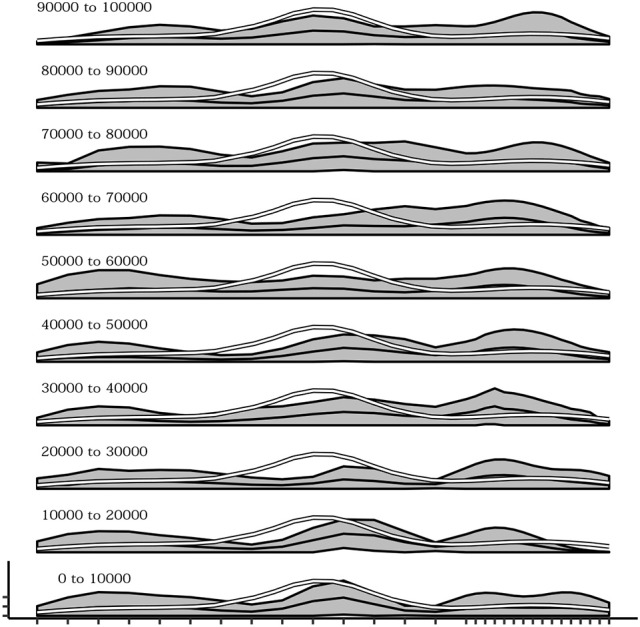
Mean activations of each sensors over several intervals (10,000 timestep) from the beginning (bottom) up to 100,000 timesteps. In the initial part of the simulation the distribution of touches tends to be greater in the right region of the body.

The influence of the density of sensors within different regions of the body during the development of self-touching behaviors was also explored. To this end, a different simulation was run in which 10 sensors (one third of the total) were uniformly distributed within the first two thirds of one dimensional body space of the agent, while the remaining 20 sensors (two thirds of the total) were uniformly distributed within the last one third of the body space. Figure 11 shows the overall effect consisting in a different distribution of the receptive fields of each sensor with respect to the standard simulations (Figure 11A—compare it with Figure 3A) and a different curve of sensor's activation means after learning in which activations are shifted to the right part of the body space (Figure 11B—compare it with Figure 3B). More importantly Figure 12 shows that during the initial phases of development (bottom plots) the means of sensor's activations is shifted to the left with respect to the standard development (see Figure 10 for a comparison) and this shifting is reverted only later on in the development.

## 4. Methods

### 4.1. Model detailed implementation

#### 4.1.1. Goal generator

**Figure 13:**
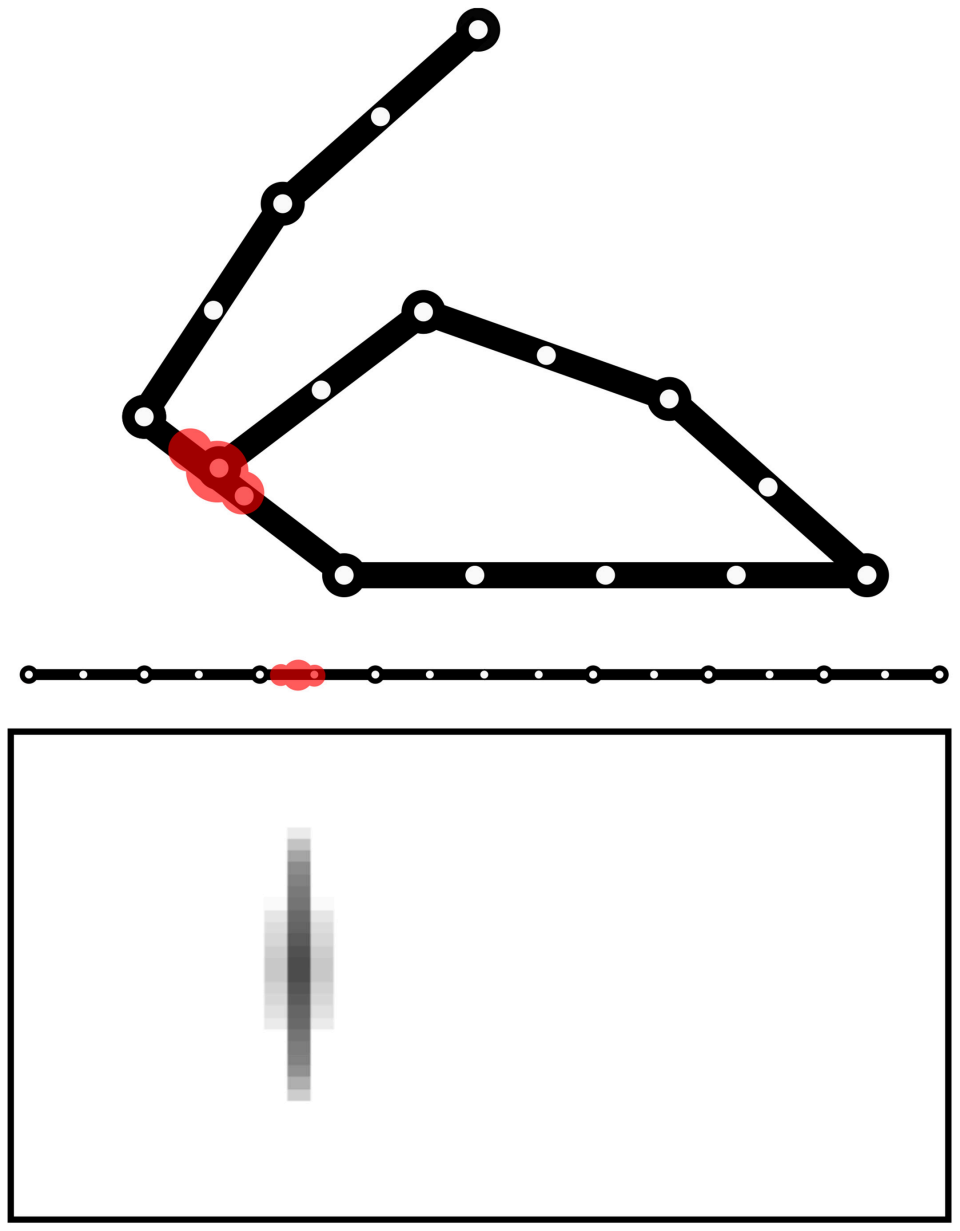
Computation of the sensory information received by the Goal Generator. The positive-change of the activation of the touch sensors on the one-dimensional body is converted into a two-dimensional neural map.

The Goal Generator performs the unsupervised formation of the abstract representations of the touch-sensor activation patterns that the system can select as goals. The activation of the touch sensors is filtered so that only the positive *changes* in the somatosensory activations are considered. The change pattern is transformed into a two-dimensional map of units where the horizontal dimension encodes the different sensors and the vertical dimension spatially encodes the activation intensity of each sensor change: this is done by determining the height of a Gaussian function used to activate the column units related to a certain sensor. Figure 13 shows this process with an example.

The Goal Generator is implemented as a self-organising map (SOM, Kohonen, 1998). SOMs are a particular kind of neural network that is able to categorise all the patterns of a given dataset in a unsupervised manner (Kohonen, 1982). Each node of the output layer of a SOM learns to detect the distance of input patterns from a prototype pattern stored in the connection weights of the unit. SOMs also acquire information about the distance between the different cluster prototypes by storing it in the n-dimensional topology of their output layer.

More in detail, we refer to the case in which the input to the SOM is a vector **x** ∈ ℝ^*n*^, and its output organised in a two-dimensional map and unrolled into the vector **y** ∈ ℝ^*m*^. Generally in SOMs each output unit *y*_*j*_ the output layer of the SOM computes the distance of the input **x** from each output-unit weight vector $w_{j}\in {\mathbb{R}}^{n}$ belonging the network connection weight matrix $W={\left[w_{1},\cdots w_{j},\cdots w_{m}\right]}^{T}$:

(1) $$\begin{array}{r} y_{j}=||x-w_{j}|{|}_{2}^{2} \end{array}$$

where $||.|{|}_{2}^{2}$ is the square Euclidean norm of a vector. The weight vector **w**_*j*_ is the prototype of the cluster represented by output unit *y*_*j*_. The best matching (“winning”) unit *y*_*win*_ is the output unit whose prototype is closest to the current input:

(2) $$win=\underset{j}{\text{arg min }}y_{j}$$

However, here the activation of the map units was computed in a different and more biologically plausible way as a standard weighted sum of the input signals, minus a bias depending on the prototype weights size. To show this, we transform the selection of the winning unit as follows:

(3) $$\begin{array}{l} win=\underset{j}{\text{arg min}}||x-w_{j}|{|}_{2}^{2}\\ =\underset{j}{\text{arg min}}({\left(x-w_{j}\right)}^{T}(x-w_{j}))\\ =\underset{j}{\text{arg min }}(x^{T}x-2w_{j}^{T}x+w_{j}^{T}w_{j}) \end{array}$$

and since the term **x**^*T*^**x** can be ignored because it is constant with respect to the minimization we have:

$$\begin{array}{l} =\underset{j}{\text{arg min }}(-w_{j}^{T}x+\frac{1}{2}w_{j}^{T}w_{j})\\ \;=\underset{j}{\text{arg max }}(w_{j}^{T}x-\frac{1}{2}w_{j}^{T}w_{j}) \end{array}$$

This leads to compute the activation of each output units as a standard weighted sum of the input minus a weight-dependent term:

(4) $$y_{j}=w_{j}^{T}x-\frac{1}{2}w_{j}^{T}w_{j}.$$

This formulation of the output layer activation has some advantages (Martín-del Brío and Blasco-Alberto, 1995), so we use it here. In particular, it is biologically plausible and allows the comparison of the units activations with a threshold (see below in this section).

The sets of connection weights reaching a given output unit *j* (unit prototype) are updated at each iteration as follows:

(5) $$\Delta w_{j}=\eta_{som}\Theta (win,j,\theta_{n})(x-w_{j})$$

where η_*som*_ is a learning rate and Θ(*i, j*, θ_*n*_) is a function of the distance of a unit *j* from a unit *i*. In the classic SOM algorithm, a threshold distance θ_*n*_ is used to define the “winning neighbourhood” that Θ = 1 if the distance of the output unit *y*_*j*_ from *y*_*win*_ within the output neural space is below θ_*n*_, and Θ = 0 otherwise. Both the distance threshold θ_*n*_ and the learning rate η_*som*_ are then exponentially decreased on each iteration so that increasingly fewer units surrounding the winning units undergo learning.

We deviate from this standard learning algorithm in one important way so as to cope with the open-ended learning nature of the architecture, where new goals can be continuously discovered, by linking the goal-formation to the competence in accomplishing them. In particular, both the learning rate *eta*_*som*_ and the neighbouring threshold θ_*n*_ are updated on the basis of the competence-based intrinsic motivation measure as follows:

(6) $$\Delta w_{j}=(1-\bar{\psi })(1-\psi_{j})\Theta (win,j,(1-\bar{\psi }))(x-w_{j})$$

where ψ_*j*_ is the competence-improvement of the SOM output unit *j* and $\bar{\psi }$ is the average of such measure for all units.

In order to compute the matching signal, the output of the SOM is filtered so that it results in a binary pattern **o** whose elements are all set to 0 with the possible exception of the element corresponding to the winner unit: this is set to 1 in the case its activation exceeds a threshold θ_*o*_.

#### 4.1.2. Goal selector

The Goal Selector is responsible for the autonomous selection of goals at the beginning of each trial. The selected-goal pattern is sent to the Motor controller to generate a movement and is also used to compute the matching signal. The Goal Selector is implemented as a layer of units **g** corresponding one-to-one to the units of the output layer of the Goal Generator. All elements of **g** are set to 0 with the exception of one element set to 1. The element set to 1 is decided on the basis of a probabilistic sampling based on probabilities computed through a softmax function getting as input the current competence improvement ξ of the goals:

(7) $$p(g_{j}|\xi )=\frac{e^{\frac{\xi_{j}}{\gamma }}}{\sum_{i}e^{\frac{\xi_{i}}{\gamma }}}$$

where γ is the “temperature” parameter of the softmax regulating how much the generated probabilities tend to favour goals with a higher competence improvement.

#### 4.1.3. Motor controller

The **g** pattern is used as input to the Motor controller sending commands to the joints of the arms. The Motor controller is formed by three components: (1) an echo-sate neural network (“dynamic reservoir network”) whose 6 output (“readout”) units encode the desired angles of the joints of the arms; (2) a structured noise that produces a random trajectory averaged with the echo-state network commands to support exploration; (3) an associative memory that learns to pair to each goal *g*_*j*_ a desired final posture causing the matching event related to the goal, and acquired by reinforcement learning: when the selected goal is matched, this posture is learned in a supervised fashion as a desired attractor-posture by the echo-state network. These components are now illustrated in detail.

##### The motor controller

Dynamic reservoirs are sparse recurrent networks that respond to inputs with dynamics that are close to chaotic behaviour, meaning that their activation is very rich but still non-chaotic (the “reservoir property,” Jaeger, 2001). Similar inputs produce similar dynamics. Moreover, when the network is fed with a constant input its activity goes through a transient dynamic activation and then settles to a stable attractor. This attractor is formed by zero values when the input is a vector with zero elements, and is formed by a certain pattern when the input is formed by a vector with some non-zero elements (different input patterns cause different attractors). The transient activation feature allows dynamic reservoir networks to learn to produce temporal sequences in output. Their convergence to attractors with constant input patterns allows them to produce movements that converge to specific postures Mannella and Baldassarre (2015). Moreover, reservoir networks have a great capacity of storing different responses to patterns because they can produce an expansion of the dimensionality of the input patterns when the number of the internal units is high with respect to the number of the input-layer units.

The units of the reservoir network used here—a leaky echo-state network—have a leaky activation potential **r** and an activation **a** as follows:

(8) $$\tau_{dr}\dot{r}=-r+W_{g\to r}g+W_{r\to r}a$$

(9) $$a={\left[tanh(r)\;\right]}^{+}$$

where τ_*dr*_ is a temporal factor, **W**_*g*→*r*_ is the matrix of weights connecting the selected-goal units **g** to the reservoir, and **W**_*r*→*r*_ is the matrix of internal connections. The initial values of **W**_*r*→*r*_ is generated with a Gaussian noise.

After being generated, the matrix has been normalised to satisfy the reservoir property (Jaeger, 2001):

(10) $$1-\epsilon <\rho (\frac{\delta t}{\tau_{dr}}W_{r\to r}+(1-\frac{\delta t}{\tau_{dr}})I)<1$$

where ρ(**M**) = *max*_*j*_(|λ_*j*_|) is the spectral radius of a matrix **M** with eigenvalues λ_*j*_, and **I** is the identity matrix.

The reservoir internal units are connected to a layer of readout units **z** setting the values of the joint angles of the arm:

(11) $$z=\;{\left[tanh(W_{a\to z}a)\right]}^{+}$$

The reservoir learning involves the weights **W**_*a*→*z*_ and is directed to produce a mapping from the selected-goal **g** received in input and the desired postures **D** produced in output and stored in the motor associative memory (see below). Indeed, **D** represents the posture experienced at the moment of the matching involving the selected goal received as input by the reservoir. To this purpose, the weights are modified *at each step* of the trial as follows:

(12) $$\begin{array}{r} \Delta W_{a\to z}=\alpha ((d_{s}-z_{t})⊙z_{t}^{\prime })a_{t}{}^{T} \end{array}$$

where α is a learning rate, **d**_*s*_ is the desired posture stored in the associative memory **D** and corresponding to the selected goal **g**_*s*_ sent as input to the reservoir, **z**_*t*_ is the output pattern of the reservoir at time step *t* of the trial and $z_{t}^{\prime }$ its element-wise first derivative, ⊙ is the element-wise product, and **a**_*t*_ is the activation of the reservoir internal units. If **d**_*j*_ has not yet been generated, as the selected goal has never been matched, learning does not take place.

##### The random trajectory generator

During learning, the output of the reservoir merged with the output of a random trajectory generator to foster motor exploration. To this purpose, at each trial the random trajectory generator produces a sinusoidal trajectory, having a frequency randomly drawn from a certain random range, for each joint *j*:

(13) $$\begin{array}{r} n_{j}=cos\left(2\pi f\frac{t}{\beta }+\pi \right) \end{array}$$

where *f* is a random frequency in the interval [0, 1] and β is a scale factor.

The final motor command issued to the joints, **m**, is a weighted sum of the reservoir output and the random trajectory generator, using as weight the competence ψ_*j*_ of the selected goal:

(14) $$m=\pi (\psi_{j}z+(1-\psi_{j})n)$$

##### The associative memory

Every time there is a matching of selected goal *g*_*j*_, the target posture associated to it, **d**_*j*_, is updated as a decaying average of the experienced postures **p**:

(15) $$\tau_{d}{\dot{d}}_{j}=-d_{j}+p$$

where τ_*d*_ is a decay factor. This factor is modulated by the competence of the selected goal:

(16) $$\begin{array}{r} \tau_{d}=\frac{1}{1-\psi_{j}} \end{array}$$

This implies that with low competence the target posture corresponding to the selected goal is strongly updated towards the experienced posture causing the accomplishment of the generated goal (corresponding to the selected goal), whereas with a high competence it freezes on its current values.

#### 4.1.4. Competence measures

This section shows how the model computes the competence for goals through the online optimization of the outcome-goal contingency prediction.

During each trial a goal matching happens (and considered equal to 1) if the Goal Generator activated unit at a given timestep corresponds to the goal selected by the Goal Selector at the beginning of the trial (otherwise the matching is considered equal to 0 at the end of the trial):

(17) $$match=o^{T}g$$

A linear neural network getting as input the selected-goal pattern predicts the goal matching (0 in the case of failure, 1 in the case of success):

(18) $$pred=\;\psi^{T}g$$

Initially, the values *ψ* are set to zero so the prediction is equal to 0. The predictions are learned to predict based on the difference between the current *match* and *pred* values:

(19) $$\Delta \psi =\eta_{pred}(match-pred)⊙g$$

Given that the range of both *match* and *pred* is [0, 1], the possible values of the elements of *ψ* tend to be in the same range. Each element ψ_*j*_ is then a measure of the competence for the goal *g*_*j*_ as, given the 0/1 values of this, it tends to represent the probability of achieving such goal when it is selected.

The model also uses the (*match*−*pred*) error to compute a second measure of competence that changes more slowly with respect to the first one by applying to it an exponentially decaying average:

(20) $$\tau_{\xi }\dot{\xi }=-\xi +\;{\left[match-pred\right]}^{+}$$

The choice of using only the positive part of the prediction error ([.]^+^) (cf. Santucci et al., 2013) is due to the fact that the intrinsic motivation signal is related to competence, thus when the system fails to accomplish a goal the leaky value (and motivation) converges towards zero rather than towards negative values. The exponential decaying average causes a slow change of the signal: as we shall see, this is important for the focussing of the system for some trials on the discovered goals and this in turn affects the convergence of the model. The parameters of the model are shown in Tables 1, 2.

**Table 1:** Parameters used in the model for all simulations.

η_*som*_	0.25
λ	0.01
τ_*dr*_	100.0
β	50.0
τ_*d*_	1.2
η_*pred*_	0.35
τ_ξ_	5.0

**Table 2:** Sizes of all the components's layers in the model.

Sensors	30
SOM inputs (pre-encoding)	20 x 20
SOM output units	5 x 5
Goal selector units	5 x 5
Motor control RNN	150
Motor control readouts	6

### 4.2. Source code

The model was developed using the Python programming language. Simulations to find the best parameters were run through the computers of the Grid'5000 system, allowing free access and use of high performance computing resources. Analyses and plots were made by using the R programming language. The source code of the simulations is available at: https://github.com/GOAL-Robots/CNRUPD_010618_sensorimotorcontingencies.

## 5. Discussion

In this work we investigated the hypothesis that self-generated goals and Intrinsic Motivations (IMs) may play an important role even in the early development of knowledge on own body and basic motor skills. This hypothesis, supported by empirical data (section 1), has been incorporated in a 2D simulated robot composed of two arms and endowed with touch sensors. The results confirm the computational soundness of the hypothesis (section 3), showing how the model is able to autonomously form a map of self-generated goals, encoded in terms of touch-sensations, and to learn the motor skills to reach the different areas of such map. The learning processes allowing the model to acquire this knowledge are completely autonomous and rely on two key processes, the autonomous generation of goals and the use of intrinsic motivations based on competence to select them.

The model autonomously generates goals based on the capacity of its movements to change own sensation, specifically, when the model discovers a contingency between a motor behavior (the achievement of a specific end-posture of the two arms) and the detection of a *perceptual change* (the activation of the touch sensors). Once generated, goals can play important functions both during learning and during functioning. During learning they can guide the refinement of the motor behavior leading to them, in this case the movements to produce the perceptual change (as in GRAIL architecture, cf. Santucci et al., 2016). In particular, the activation of the *internal representation* of a goal allows the model to learn the motor skill to accomplish it independently of the fact that the contextual input from the environment, here the possible states of own body, is always the same. This would not be possible within a stimulus-response reactive framework, e.g., with standard reinforcement learning models (Sutton and Barto, 1998), as the constant context (“stimulus”) would not allow the system to perform different motor behaviors. Instead, the model can learn different motor behaviors as it can associate them to different internally-activated goals. Moreover, goals support a second function during learning, namely the generation of a “matching signal,” produced when the experienced sensation (here the touch sensation) matches the internally-activated goal representation: such signal produces a reward that guides the trial-and-error learning process supporting the refinement of the motor skill directed to pursue the currently-active goal.

During functioning, goals can serve the role of “pointers” to recall the acquired skills. Indeed, the activation of a goal can trigger the performance of the motor skill that accomplishes it even if the context has no change. Again, reactive models cannot do this as they cannot recall different skills unless information from the outside is provided (e.g., in the form of a pointer somehow associated to each skill). Here the activation of goals to test this functionality of the model is done by hand but in the future the enhancement of the model within a developmental framework might endow it with the capacity to autonomously employ goals to recall the related motor skills to accomplish similar desired goals or to facilitate their learning (Seepanomwan et al., 2017), or to compose more than one goal/skill to form more complex policies accomplishing goals at a coarser granularity (Vigorito and Barto, 2010; Hart and Grupen, 2011).

The second important process guiding the body knowledge acquisition in the model is related to competence-based intrinsic motivations linked to the acquisition of the motor skills leading to the desired goals. This motivation is computed on the basis of a mechanism measuring the probability that a skill accomplishes the goal to which it is linked. As it typically happens in intrinsic motivations, these mechanisms are related to the acquisition of information (in this case the capacity to reach own body) and has a transient nature (Baldassarre, 2011), i.e., it leads to decrease the agent's interest in an activity when the competence in that activity has been acquired. In the model, competence-based intrinsic motivations plays several different functions. First, a low competence favors the update of the representations of goals whereas a high competence leads to stabilize them. Second, the opportunity to gain competence guides the selection of the goals on which the agent focuses its exploration and learning resources. Third, high levels of competence for a goal reduce motor noise used to search the motor behavior to accomplish it. Fourth, a low competence for a certain goal leads to a substantial update of the related movement target (and hence of the related movement) whereas a high competence leads to its stabilization. Overall, when integrated these mechanisms lead the agent to converge to stable action-outcome contingencies, namely to both effective movements to accomplishing goals and to stable goal representations.

The dependence of the autonomously formed goal representations on competence is particularly innovative. The introduction of the dependence of goal representations on the competence to accomplish them was a critical step that allowed the model to be able to form stable goals and skills, with goals covering the whole body space in a homogeneously distributed fashion. To our knowledge, this is the first work that uses competence-based intrinsic motivations to modulate the formation of the perceptual representations related to goals, and to show its importance for the overall stability of the discovered action-outcome (“skill-goal”) contingencies.

In the computational literature, other works proved the power of self-generated goals and IMs to boost the autonomous learning of knowledge and competences. While the majority of these works focused on the acquisition of some sort of control on the environment (e.g., Vigorito and Barto, 2010; Santucci et al., 2014a; Kulkarni et al., 2016; Forestier et al., 2017; Seepanomwan et al., 2017), here we wanted to test how similar principles could be used to drive the learning of low-level motor skills based on the interaction with own body. In the goal-babbling literature, some works (e.g., Rolf et al., 2011; Baranes and Oudeyer, 2013; Rolf and Steil, 2014) use the autonomous generation of intrinsic goals to learn a mapping between different end-points in the goal space and the corresponding configuration of the redundant effectors of the robots. Differently from these systems, our model is able to jointly map two different dimensions of the agent: the proprioception of its arms (the postures) and the activation of the touch sensors, thus providing a more sophisticated learning of the contingencies related to the agent's body.

Another relevant computational framework regards *knowledge gradients* (Frazier et al., 2008; Scott et al., 2011; Wu et al., 2017). This is a Bayesian method to optimize the exploration of some alternative options, each carrying a stochastic reward, based on the information-gain gradient related to them and with the objective of a later choice of the best option. Our model has some similarities with the idea of knowledge gradients since it uses a value function over the space of the internal representations of goals to bias their selection. The major difference is that in our model the knowledge gain giving rise to competence-based IM concerns the competence of the motor controller. Instead, in knowledge gradients the knowledge gain regards the increase of the confidence of the estimate of the rewards of options. Moreover, in our model the value function for goal exploration is built upon a “utility function” that is non-stationary, namely the competence of goal which varies with motor learning, whereas knowledge gradients are built upon a value function related to the information gain concerning the esteem of the rewards of alternative options which are fixed. Given these similarities, an interesting line of research would be to cast our hypothesis within a probabilistic framework such as that of knowledge gradients, where the exploration is guided by a measure of competence gain computed through Bayesian optimization.

The hypothesis that goals might be used also to learn low-level fine-grained motor skills is in agreement with evidence from neuroscience. This shows that, alongside high-level goals encoded in the prefrontal cortex (Miller and Cohen, 2001; Mannella et al., 2013), premotor and motor cortical areas might encode movements in terms of goals related to desired end-movement postures (Graziano et al., 2002) or body-object relations (Umilta et al., 2001).

Empirical evidence from developmental psychology relevant to the present model of the acquisition of self-touch behavior is not plentiful. However some evidence can be summoned at a more general level from experiments showing the role played by sensorimotor contingencies in development. In particular, it has been shown that contingencies related to producing relevant changes in the environment, in particular the movement of a mobile toy attached with a ribbon to one arm of a baby but not to the other arm, can lead to an increase movement of the relevant arm (Rovee-Collier et al., 1980).

More recently it has been shown how learning progress related to reaching a toy can be enhanced by the fact that the object produces a sound contingently to its touching (Williams and Corbetta, 2016). Overall this evidence indicates the importance of contingencies for the development of motor skills, and in particular of the fact that actions lead to a *change* in the world. This is also a key assumption of the model, although it remains to be ascertained by the use of appropriately designed empirical experiments if those contingencies drive the learning of motor skills through the mediation of goal-formation, as proposed here, or directly within a stimulus-response framework.

Another source of relevant research concerns the development of reaching toward the own body and toward objects. This research shows that infants develop progressively from spontaneous to goal-directed arm and hand movements. Wallace and Whishaw (2003) examined hand movements in infants aged 1–5 months and describe a development from closed fists to open hand movements that progress in complexity toward self-directed grasping. Such “hand babbling” may correspond to the goal-directed babbling of the models behavior. Also, about a month before infants execute their first successful reaches, they increase the number of arm movements in the presence of a toy and raise their shoulders and arms in approximation of a reach (Bhat and Galloway, 2006; Lee et al., 2008), which again suggests that spontaneous arm movements or “arm babbling” prepares the emergence of purposeful reaching. Thomas et al. (2015) documented self-touching behavior in developing human infants over the first 6 months of life. In the initial weeks, they mainly observed movements around the shoulders with the digits in a closed fist configuration, resulting in incidental contacts with the body. From about 12 weeks, movements included palmar contacts, giving a goal-directed, exploratory quality to self-touch.

We shall now review how more specific predictions of the model may be linked to existing data in developmental psychology as well as the perspectives that these predictions open for future research. One first prediction from the model is that movement duration should decrease with learning progress, in particular for specific goals, as the competence to accomplish them increases. Certainly it is true that infants' reaches become smoother and straighter over development. First reaches have irregular, inefficient, curved paths, with several changes in direction and multiple bursts of speed, making the path up to four times longer than a straight line to the object (von Hofsten, 1991). Evidence from infants reaching in the dark shows that this initial inefficiency is not due to continuous visual tracking of the hand relative to the target in a series of corrections. In fact, infants produce similar hand paths and reach characteristics when reaching for glowing objects in the dark (Clifton et al., 1991). It is therefore possible that the inefficient movement phase reflects motor babbling. Similarly, between 6 and 15 months of age, infants' arm movements while banging a block or wielding a hammer become increasingly straight and efficient (Kahrs et al., 2013). We are currently investigating how reaching toward vibrotactile targets on the infant's own body develops between 4 and 6 months of age. Based on the model we expect that reaches toward locations on the body will gradually become faster and more efficient.

A second, obvious, prediction of the model is that skill acquisition should progress from easy to hard skills. In our series of observations of self-touch, we expect to observe a developmental sequence of reaching for parts of the body that are easier to attain, such as the mouth or the hips, toward reaching for targets that are more difficult to find, such as the forehead or the earlobes. Our current investigations with infants between the ages of 2 and 6 month along with the results reported by Chinn et al. (2017) with older infants appear to confirm this developmental trend.

Finally, a third prediction from the model regarding human development holds that uneven density of tactile receptors throughout the body should contribute to determining which areas of the body are contacted earlier. This prediction is partially confirmed by existing empirical data that shows that infants' and fetuses' first self-touch behaviors involve areas with high tactile receptor density such as the mouth or the thumb (De Vries et al., 1982). These regions also produce approach motions of the hands which have faster dynamics as compared to other regions (Zoia et al., 2007). It should be noted however that alternative models, such as that designed to account for fetus behavior by Mori and Kuniyoshi (2010) may make similar predictions.

## Author contributions

FM, VS, GB designed the model and the computational framework. FM carried out the simulation and the analysis of the results. KO, ES, LJ contextualized the computational model within the framework of empirical literature. FM, VS, ES, LJ, KO, GB contributed to the writing of the paper.

### Conflict of interest statement

The authors declare that the research was conducted in the absence of any commercial or financial relationships that could be construed as a potential conflict of interest.
